# Advanced preclinical testing of a design-optimized ceramic bearing for a cervical total disc replacement

**DOI:** 10.1371/journal.pone.0339851

**Published:** 2026-02-05

**Authors:** Lucia Kölle, Gregory Pryce, Andrew R. Beadling, Michael Bryant, Richard M. Hall, Stephen J. Ferguson, Benedikt Helgason

**Affiliations:** 1 Department of Health Sciences and Technology, ETH Zürich, Zürich, Switzerland; 2 Institute for Functional Surfaces, School of Mechanical Engineering, University of Leeds, Leeds, United Kingdom; 3 Department of Mechanical Engineering, School of Engineering, University of Birmingham, Birmingham, United Kingdom; VIT University, INDIA

## Abstract

**Objective:**

Arthroplasty in the cervical spine is performed using Total Disc Replacement (TDR) implants. This study investigates the safety and tribology of a design-optimized ceramic TDR bearing.

**Methods:**

Samples of an optimized bearing geometry were manufactured from the bioceramic BIOLOX^®^*delta* (CeramTec GmbH, Germany) and tested in a six degree of freedom universal joint simulator in diluted calf serum at body temperature. The investigated load and motion profiles simulated movements of daily living; three pure rotations under static load and three rotations coupled under sinusoidal load as well as adverse events (three subluxation test conditions).

**Results:**

Dynamic coefficients of friction during flexion/extension and lateral bending were µ = 0.16 ± 0.011 and µ = 0.16 ± 0.070, respectively. Friction factors during flexion/extension and lateral bending were f = 0.14 ± 0.0092 and f = 0.13 ± 0.063, whereas the friction factor in axial rotation was f = 0.033 ± 0.0028 and for three coupled rotations under sinusoidal load, it was f = 0.13 ± 0.028. The design demonstrated a sufficient resistance against subluxation, with subluxation forces more than 3.5-fold the 20N that are typically required as acceptance criteria in FDA safety and effectiveness data.

**Conclusion:**

Friction values were found to be comparable to or higher than those typically reported for ceramic-on-ceramic hip replacements. Furthermore, the results indicated that the optimized bearing design is sufficiently resistant to subluxation. *Significance:* Test protocols to investigate safety and tribology of cervical TDRs are proposed and their results are reported for a design-optimized ceramic TDR bearing.

## 1. Introduction

Neck pain is a common physical complaint, from which over 300 million people suffered for more than three months in 2015 [1]. When myelopathy, radiculopathy and/or neurological deficits cannot be treated conservatively within a reasonable timeframe, surgical intervention may be considered. Two standard options are spinal fusion, where the vertebrae of the affected spinal level are rigidly connected to each other and arthroplasty with a Total Disc Replacement (TDR) that aims to preserve segmental motion. TDR-reoperation rates within 7 years of follow-up are about 5% at the index level and about 4% in adjacent levels, which is about half the rates reported for fusion treatments, which are 13% at the index level and 11% in adjacent levels [2]. This difference in reoperation rates is a considerable contributor to cervical arthroplasty generating less costs than fusion over a 7 year timespan, when treating symptomatic degenerative disc disease [3]. TDRs reoperation rates are however still considerable and should be minimized. The complications underlying these reoperations are connected to a multitude of factors, some of which are related to implant design, material, and/or tribology.

Besides the classical ball-and-socket type TDRs, there are, and were, also devices of other design types on the market [4,5]. These implants often offer not only rotational, but also additional translational motion and therefore potentially a closer match to the natural kinematics of an asymptomatic spinal segment. Especially in devices with non-congruent bearing surfaces, the tribology and response to adverse loading could differ from that of ball-and-socket TDRs.

As safety and efficacy are of utmost importance for any implant, device function must be investigated, especially to identify deficits such as elevated friction and risk of subluxation. TDR design parameters such as the sphere-radius can affect the lubrication regime and the resulting friction coefficients [6], therefore experimental assessment of the design is required. It is furthermore necessary to evaluate the response of the TDR to adverse loading conditions, for which it may not be optimized. Excessive or minimal friction might limit the spinal structures’ ability to guide motions close to natural kinematics. This in turn might affect other issues such as adjacent segment degeneration. Currently, literature on tribological evaluation of TDRs and on subluxation tests of TDRs is scarce.

Ceramics are a promising material choice for TDRs [7,8]. Compared to other typical implant materials, they have high wear resistance, excellent biocompatibility, high strength and imaging compatibility [7]. In a previous study [9], we designed a ceramic TDR bearing aiming to minimize the risk of hypermobility while enabling motion-coupling. The design was optimized under coupled flexion/extension-anterior/posterior translation motion intending to replicate a realistic scenario. Nevertheless, there are performance aspects that are challenging to model *in silico*, such as friction, which could prevent the use of such a design of ceramic-on-ceramic articulation surfaces. There is a general consensus that the primary cause of squeaking is friction [10]. To assess whether these potential improvements are achieved at the expense of other measures of functional performance, we conducted *in vitro* simulator testing of the safety and tribology of the design optimized ceramic ball-in-trough bearing. The aim was to experimentally determine whether a computationally optimized ceramic TDR would demonstrate adequate function and safety under loading conditions for which it was not optimized. We hypothesized that the friction parameters based on literature values for BIOLOX®*delta-*on*-*BIOLOX®*delta* hip replacements are representative of the presented TDR design and that the optimized ceramic bearing has sufficient resistance to subluxation.

## 2. Materials and methods

In the present study, the safety and tribology of a ceramic ball-in-trough TDR bearing [9] intended for the lower cervical spine, particularly the C6/C7 level, are investigated. Specifically, friction in isolated flexion/extension, lateral bending, and axial rotation as well as in coupled rotations was investigated, and subluxation forces were measured during anterior, lateral and posterior subluxation.

### 2.1. Sample preparation

**Fig 1:**
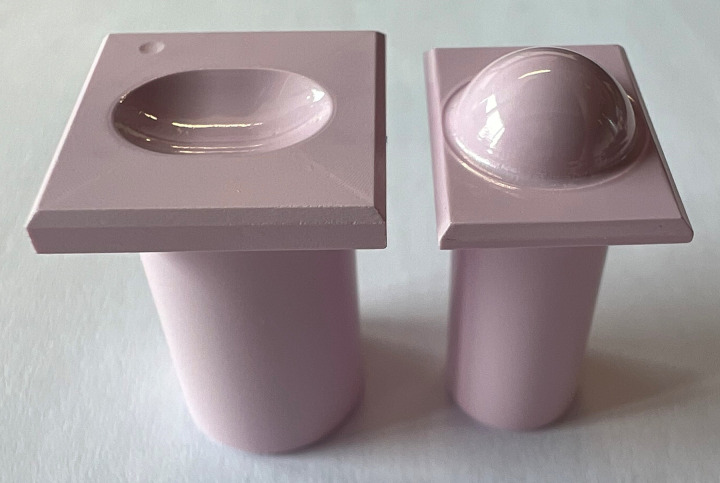
Ceramic sample. Left: inferior part, right: superior part. The small deepening on the inferior part (on the top left in this picture) marks the posterior side.

**Fig 2:**
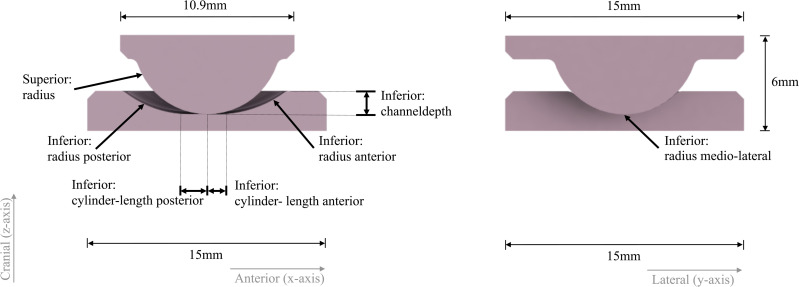
Schematic representation of the bearing geometry parameters. Left: sagittal cross section, right: coronal cross section.

**Fig 3:**
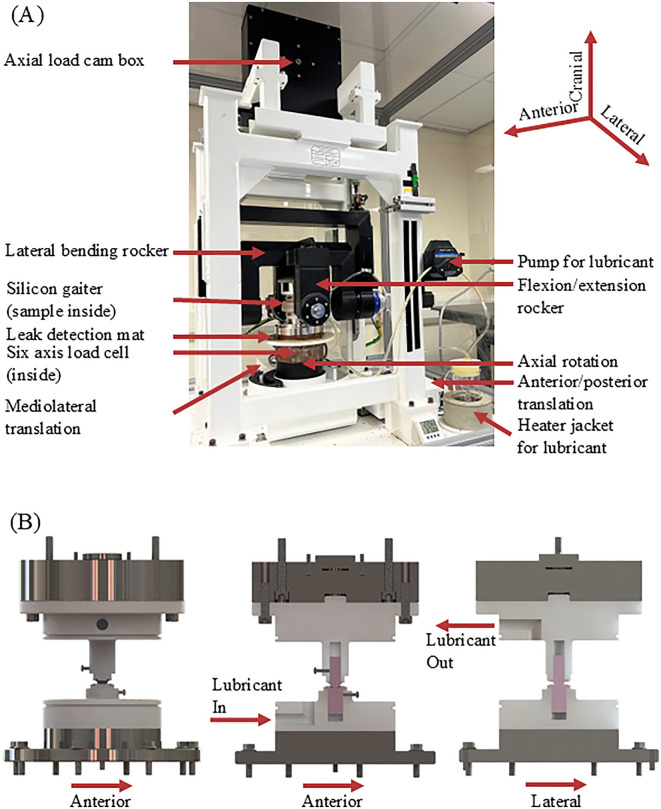
Universal Joint Simulator used for testing of the TDR bearing. Upper image (A): Testing arrangement. The silicone gaiter is fixed to the polyoxymethylene fixtures. The coordinate system refers to the sample, the rotations can be understood based on that and from the mechanical setups rotation axes. Lower images (B): Computer Aided Design renderings of the custom fixtures for the universal joint simulator with a test sample in place. Assembly view (left), sagittal (centre) and coronal (right) cross sections. The grey parts are made from stainless steel; the light grey parts are made from polyoxymethylene. The silicone gaiter is not shown for clarity. The ceramic samples are shown in pink. The sphere centre of the superior part of the bearing is in the centre of rotation of the simulator in all three axes.

Samples were manufactured featuring a bearing geometry (Figs 1 and 2) resulting from a design optimization that is described in detail in a previous study [9]. These samples were manufactured from the alumina matrix composite ceramic BIOLOX®*delta* (CeramTec GmbH, Germany) including specific manufactuing principles from well established hip and knee bearings. The bearing surfaces were hand polished. An example of the finished samples can be seen in Fig 1. The cylinders and rectangular endplates serve to securely and precisely fix the samples in the fixtures of a six-degree-of-freedom universal joint simulator (Fig 3). Prior to testing in the universal joint simulator, the samples were cleaned in an ultrasonic bath (VWR, Ultrasonic Cleaner USC – TH, VWR International, USA) in distilled water for 10 minutes and dried with an airgun (Legris, Parker Hannifin Corporation, USA). Right before testing, the articulating surfaces were cleaned with acetone and lab wipes.

The geometries of the manufactured samples (Fig 2) were measured using a contactless ATOS III Rev.02 SO MV100 (Carl Zeiss GOM Metrology GmbH, Germany) for comparison to the designed geometry and for the evaluation and interpretation of the universal joint simulator measurements. Contactless surface roughness analysis was performed using Vertical Scanning Interferometry (VSI) using a NPFLEX (Bruker Corporation, USA) under a 50x objective to obtain areal roughness values. A procedure was established to perform the scans on the same positions on each sample. Scans were performed on the centres of the articulating surfaces, as this was where the contact point of the implant parts was expected to be most during the tests. Before scanning, the bearing surfaces were cleaned using acetone and lab wipes. The software Vision64™ (Bruker Corporation, USA) was used for the evaluation. Form removal was done (superior sample part: for curvature and tilt, inferior part: cylinder and tilt) to account for the samples bearing geometry; peripheral data was masked as the scan quality further away from the centre of the scan is not as high. On the inferior part, masking left a 0.5x0.5mm square for evaluation. On the superior part, masking left a 0.5 mm diameter circular area for the evaluation. A second order Gaussian regression filter was applied to remove waviness. Second order was selected as the bearing surfaces are highly curved, a long wavelength cut-off at 0.08 mm was used based on [11]. Areal surface roughness (S_a_) was evaluated.

### 2.2. Universal joint simulator testing

All testing was performed using a six degree of freedom universal joint simulator (Prosim Single-Station Universal Simulator, Simulation Solutions Ltd, UK) (Fig 3), that can exert up to 11kN of axial load, between +40° and −120° of flexion/extension, + /-40° in axial rotation and lateral bending, + /-25 mm in mediolateral translation or up to 1.5kN in this direction, + /-25 mm in anterior translation or up to 1.5kN in this direction; execute motions between 0.01 Hz and 2.5 Hz and all of its axes can be used in force and displacement control [12]. Within the simulator, the specimens were submersed in calf serum, diluted to a concentration of 20g ± 2g protein/l based on ISO 18192–1:2011 [13] (serum: GE Healthcare Lifesciences, HyClone Laboratories, USA) which was kept at 37°C (+/-2°C) throughout the tests. Sodium azide (Severn Biotech Ltd., UK) was added at a concentration of 0.03% to inhibit bacterial growth. Custom fixtures (Fig 3) kept the lubricant within a silicone gaiter and the sample fixed in place and aligned. A pump circulated the lubricant constantly, a heater jacket maintained the desired temperature of the lubricant. The universal joint simulator’s”Spine Machine” mode was selected to adjust the axis definitions, as the present study concerns a bearing for a spinal implant.

Each sample was tested under multiple load- and motion profiles (see Table 1). Between tests, the universal joint simulator was repositioned via the software to create a distance between the two ceramic parts and the load readings were set to zero.

**Table 1:** Test profiles used for investigation of the optimized ceramic bearing in vitro in a simulator.

Test profile	Axial load	Motion	Frequency/ Motion speed	Samples	Cycles	Based on/ Inspired by
**Flexion/** **extension**	Static 100N	+/- 7.5°	1 Hz	n = 5	200	ASTM F2423 – 11 [15]
**Axial rotation**	Static 100N	+/- 6°	1 Hz	n = 5	200	ASTM F2423 – 11 [15]
**Lateral bending**	Static 100N	+/- 6°	1 Hz	n = 5	200	ASTM F2423 – 11 [15]
**Three coupled rotations under sinusoidal load**	50-150N (sinusoidal)	Flexion/ extension: + /- 7.5°; lateral bending: +/- 6°; axial rotation: +/- 4°	1 Hz	n = 6	200	ISO18192 [13]
**Subluxation: anterior with parallel endplates**	Static 100N	6.5mm anteriorly	6 $\frac{mm}{min}$	n = 5	10	Simplify FDA SSED [16]
**Subluxation: posterior with 12° extension**	Static 100N	6.5mm posteriorly	6 $\frac{mm}{min}$	n = 5	10	Simplify FDA SSED [16]
**Subluxation: laterally with parallel endplates**	Static 100N	5mm laterally	6 $\frac{mm}{min}$	n = 5	10	Simplify FDA SSED [16]

The motivation for the definition of the individual test profiles was to investigate function under typical conditions and safety under extreme conditions. To measure friction values during movements the TDRs would perform most in later use in patients, the individual main rotations flexion/extension, lateral bending, and axial rotation were selected. To consider that these motions are typically performed in coupled motions in vivo [14], a load- and motion profile of three coupled rotations was tested as well. The uncoupled motions allow comparison to more literature and to investigate the behaviour without coupling effects. As subluxation could be a main concern of the investigated design, which has a rather low trough depth, three subluxation test profiles were investigated.

The samples were tested under flexion/extension, lateral bending, axial rotation and coupled rotations for 200 cycles each and the subluxation tests were carried out for 10 cycles each. Coupled rotations were tested on 6 samples, the other profiles on 5 samples (one sample was damaged due to operator error during testing).

For flexion/extension, lateral bending, axial rotation and coupled rotations, the 180^th^-189^th^ cycle (out of 200 cycles) of each experiment were evaluated. In case of the subluxation tests, the eighth cycle (out of 10 cycles) of each experiment was evaluated. This was done to avoid run-in and run-out effects. To correct for sensor drift and misalignment in each friction experiment, mean values of the simulator’s force and torque outputs (except axial load) for each individual cycle were subtracted from the measurements for each individual sample. Standard deviations represent comparisons between the samples.

There are two dimensionless factors commonly used to describe dynamic friction in biotribology. While the coefficient of friction is more commonly known, it was already customary in 1981 to report friction factors in hip joint studies [17] and this factor has found use in spine [18], shoulder [19] and knee [20] replacement studies as well. Typically, the coefficient of friction is calculated using a friction force, whereas the coefficient of friction is obtained via a frictional torque. The friction factor has the same value as the coefficient of friction only in point loading (point contact) conditions [21,22]. Friction factor and coefficient of friction become more similar when the clearance is high and the materials moduli are high [22]. The pressure distribution could be affected by congruence which in turn may be affected by radial clearance and design type.

In case of ceramic joint replacements, the coefficient of friction is commonly used, likely because the rigid smooth surfaces are usually tested under moderate loads, therefore deformation and adhesion do not have to be considered, and the coefficient of friction might be considered a constant value that is independent of the load applied. This makes it suitable for comparing values reported for different applications. We report the coefficient of friction as it is commonly used in finite element simulations, making it valuable for the computational biomechanics research community, and to compare our results to those of other studies. We additionally report friction factors for comparability to further studies.

#### 2.2.1. Flexion/extension, lateral bending and axial rotation.

Under 100N static axial load, sinusoidal rotational profiles of either up to +/- 7.5° flexion/extension, + /- 6° lateral bending or +/- 6° axial rotation, were applied. This was based on the ASTM F2423 – 11 [15], but in case of flexion/extension and lateral bending the load was applied in the global coordinate system instead of perpendicular to the superior TDR-endplate as the simulator could not accurately produce the small loads that force decomposition would have required. The test frequency was 1 Hertz which is in line with the ASTM standard.

While both static and dynamic coefficients of friction exist, in this manuscript we will refer to the dynamic one, unless otherwise specified, for simplification. The coefficients of friction in the flexion/extension and lateral bending tests were determined as follows. The 20% of the cycle around 0° rotation were evaluated to obtain the dynamic coefficient of friction µ $=\frac{F_{F}}{F_{Ax}}$, where $F_{F}$ is the friction force and $F_{Ax}$ is the axial force. The friction force being the anterior-posterior force in case of flexion/extension and the medio-lateral force in case of lateral bending. The inner 20% of the motion profile (around zero-degree rotation) was chosen to capture the kinetic friction process. Specifically, this means that the dataset between −1.5° and 1.5° rotation is evaluated in case of flexion/extension and between −1.2 and +1.2° rotation in lateral bending. This is a standard procedure in arthroplasty tribology, and the reason for this can be seen in Fig 5. When the movement direction changes, the maximal friction force is measured as the movement in the new direction starts (static friction). This behaviour connected to starting movements should not be part of the evaluation of the dynamic coefficient of friction.

**Fig 4:**
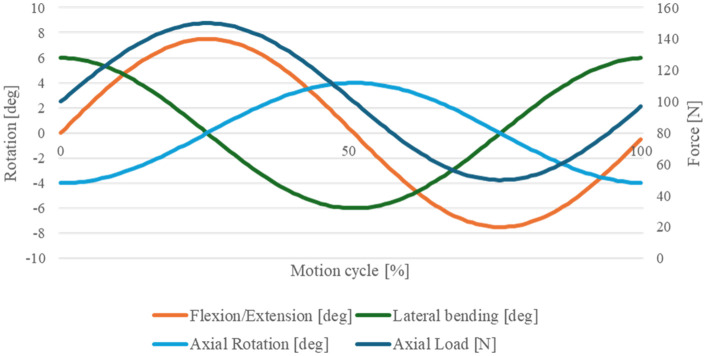
Load- and motion profile for the test profile: three coupled rotations under sinusoidal load. It is based on the wear test standard ISO18192 for cervical TDRs.

For a more thorough comparison to literature on friction in joint replacements, the friction factor was calculated as well. The friction factor was calculated as $f=\frac{T_{F}}{{l*F}_{Ax}}$ with l being the lever arm of the friction force [23] which is the distance between the centre of the loadcell and the contact point between the implant parts and $T_{F}$ the frictional torque. In the case of flexion/extension, the friction torque was the torque in flexion/extension $T_{FE}$, in case of lateral bending, the lateral bending torque $T_{LB}$ was used and in case of axial rotation, it was the axial rotation torque $T_{AR}$. The 20% most central portion around the starting position (zero degrees of rotation) in the test cycle was evaluated to capture the kinetic friction process. While there are publications related to the hip that calculate the friction factor as $f_{r}$ $=\frac{T_{F}}{{r*F}_{Ax}}$ [24] with r being the ball radius, in the present study the lever arm of the friction force here does not equal the ball radius and therefore this would not be mechanically accurate.

The differences in equations used in test evaluation correspond to differences in experimental setups and implant designs. It is crucial to define such equations based on the specific experiment rather than indiscriminately adopt methods from literature that might not be applicable for the investigated experiments. After isolating the central 20% of the cycle, force or torque values (for coefficient of friction or friction factor) were separated by sign and, for each datapoint, the friction value was calculated using the individual datapoint’s axial force. Then, the mean of the absolute positive and negative friction values was calculated.

For axial rotation, the coefficient of friction could not be calculated using a force tangential to the normal force, therefore only the friction factor was calculated. The reason is that the frictional effect in that case is of rotational nature, a frictional torque.

#### 2.2.2. Three coupled rotations under sinusoidal load.

The wear test standard ISO18192 [13] defines a load- and motion profile for cervical TDRs consisting of a coupled motion of flexion/extension, lateral bending and axial rotation under a sinusoidal axial load, see Fig 4. The frequency of 1 Hertz is given in the ISO standard.

The friction factor during this test was calculated based on the methodology used in previous work on hip replacements [25]. The overall frictional torque was calculated as:


$$T_{overall}=\sqrt{{T_{FE}}^{\text{2}}+{T_{LB}}^{\text{2}}+{T_{AR}}^{\text{2}}\;}$$

based on which the friction factor was calculated as:

$f=\frac{T_{overall}}{l*F_{Ax}}.\;$ The literature study used for orientation on the friction factor evaluation [25] used the sphere radius, but as explained before, the present study uses the distance l. Also, their calculation of the overall frictional torque is more complicated, reflecting their more complex experimental setup. This reflects the different applications these setups model. Their hip joint setup holds the sample at a physiologic orientation, while in case of our implant for the lower cervical spine, we can assume the segmental angle to be zero.

#### 2.2.3. Subluxation.

The subluxation tests were inspired by the Simplify^®^ (Simplify Medical, USA) FDA Summary of Safety and Effectiveness (SSED) test description [16]. Under 100N static axial load, displacement was applied at 6 mm/min up to 6.5 mm (a) anteriorly with the endplates parallel, (b) posteriorly with the endplates at 12° extension and (c) up to 5 mm laterally with the endplates parallel. For this, the inferior part of the sample was moved (a) posteriorly, (b) anteriorly and (c) laterally. The subluxation force was determined as the absolute value of the maximal or minimal force during each subluxation test. The main motivation to investigate lateral subluxation was to compare the behaviour of the different geometrical aspects. In the Simplify SSED, 100N axial preload and 6 mm/min anterior load were applied to one endplate in parallel position, and in 12° extension.

## 3. Results

### 3.1. Sample geometry and surface roughness

The radial clearance of the samples was 0.24 ± 0.05 mm (mean ± SD) compared to 0.07 mm in the optimized design. Further geometrical measurements and how they relate to the optimized geometry are provided in Fig 2 and Table 2.

**Table 2:** Geometric values. Mean values refer to all six samples used in the tests though some tests only used five of them.

	Optimized design [mm]	Average sample values ± SD [mm]	Difference between average of samples and optimized design [%]
**posterior radius**	6.50	6.48 ± 0.04	−0.26
**anterior radius**	6.98	6.89 ± 0.04	−1.41
**medio-lateral radius**	4.68	4.82 ± 0.05	3.16
**channel depth**	1.46	1.46 ± 0.01	0.33
**superior radius**	4.61	4.59 ± 0.003	−0.41
**posterior cylinder length**	1.16	1.17 ± 0.07	1.22
**anterior cylinder length**	0.68	0.74 ± 0.06	8.62

Surface roughness assessment showed the average roughness of the superior part was S_a_ = 24.5 ± 2.89nm. For the inferior part, it was S_a_ = 34.7 ± 13.1 nm.

### 3.2. Universal joint simulator testing

No squeaking was audible during any of the tests. No dislocations occurred, except for subluxation testing in which dislocations were enforced. The main findings of the present study are summarized in Table 3 and thereafter more details are given.

**Table 3:** Main results of testing.

Test profile	Main results (mean ± SD)
**Flexion/extension**	Dynamic coefficient of friction: µ = 0.16 ± 0.01; Friction factor: f = 0.14 ± 0.009
**Axial rotation**	Friction factor: f = 0.033 ± 0.003
**Lateral bending**	Dynamic coefficient of friction: µ = 0.16 ± 0.07; Friction factor: f = 0.13 ± 0.06
**Three coupled rotations** **under sinusoidal load**	Friction factor: f = 0.13 ± 0.03
**Subluxation:** **anterior with parallel endplates**	Subluxation force: 88.82 ± 4.08N
**Subluxation:** **posterior with 12° extension**	Subluxation force: 77.46 ± 2.4N
**Subluxation:** **laterally with parallel endplates**	Subluxation force: 103.57 ± 7.59N

#### 3.2.1. Flexion/extension, lateral bending and axial rotation.

**Fig 5:**
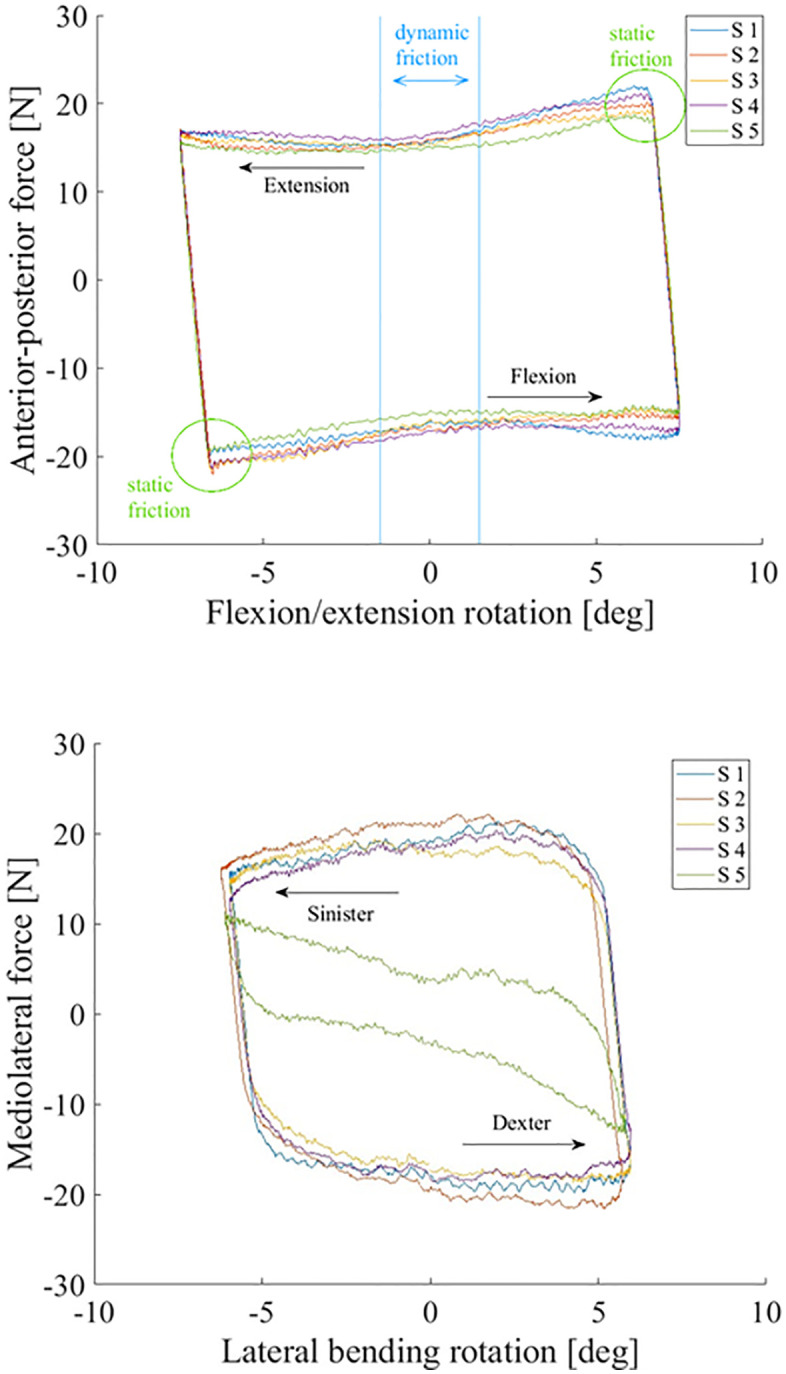
Friction loop of the flexion/extension test (above) and lateral bending test (below). The movement cycle starts at the bottom centre of the loop. The flexion movement profile starts with flexion (positive rotation), while the lateral bending movement profile starts with bending to the right side (dexter) (positive rotation). The movement direction is indicated by the arrows. In the flexion/extension test data, it can be seen, that when the movement direction changes and the movement into the new direction starts, higher friction (static friction) is measured, then in the central part of the loop (dynamic friction). The datapoints between the blue vertical lines were included in the evaluation of the dynamic friction. The green circles show where static friction could be evaluated. Such friction hysteresis loops are also used in studies on fretting wear, Fantetti et al. give a detailed description of these loops [26]. S = Sample.

The dynamic coefficients of friction during flexion/extension and lateral bending were µ = 0.16 ± 0.011 (mean ± SD) and µ = 0.16 ± 0.070 (mean ± SD) respectively. Fig 5 provides more detail on the test results.

The friction factors were f = 0.14 ± 0.0092 (mean ± SD) for flexion/extension and f = 0.13 ± 0.063 (mean ± SD) for lateral bending.

**Fig 6:**
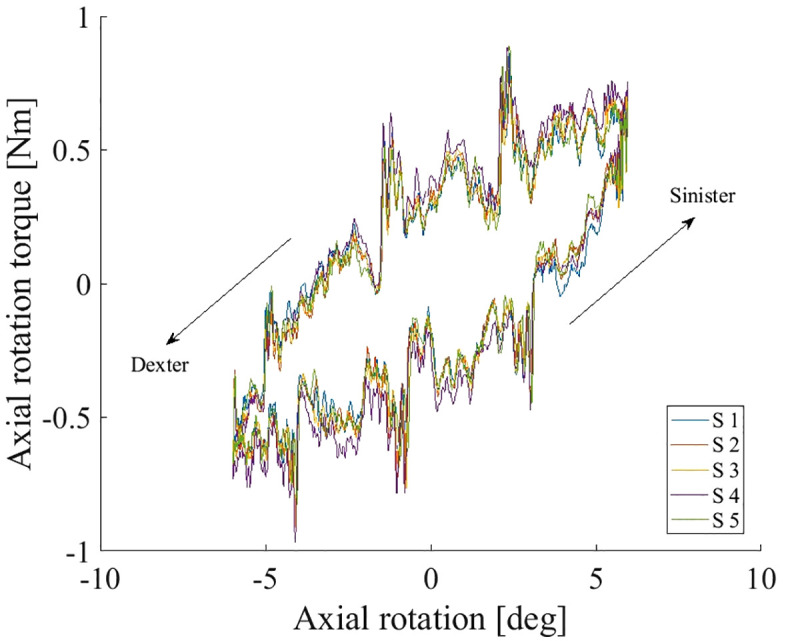
Axial rotation torque over axial rotation in the axial rotation test. The movement cycle starts at the bottom centre of the loop and then moves toward positive rotation initially (the inferior part of the simulator rotated to the right side thereby simulating axial rotation to the left). The arrows indicate the movement direction.

The friction factor during axial rotation under a constant load was f = 0.033 ± 0.0028 (mean ± SD). Fig 6 shows the axial torque over axial rotation.

#### 3.2.2. Three coupled rotations under sinusoidal load.

**Fig 7:**
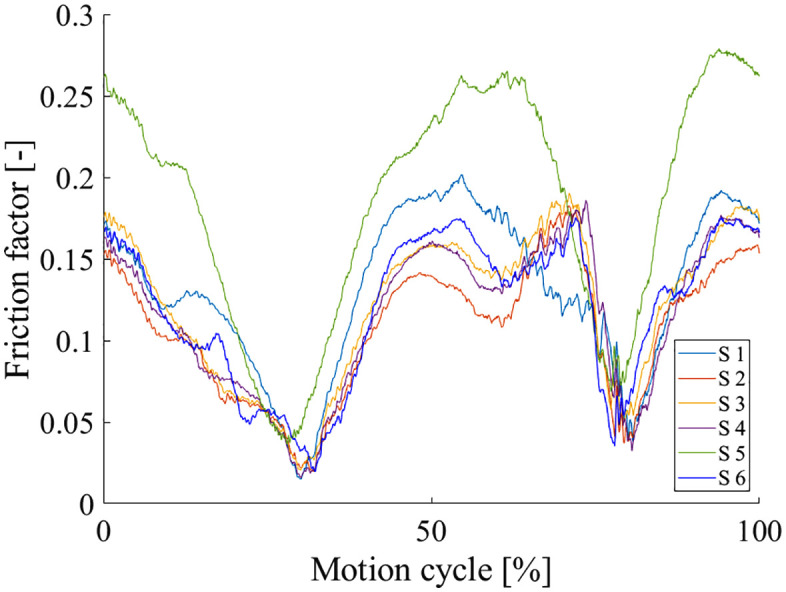
Mean friction factor over the course of the test cycle, during three coupled rotations under sinusoidal load. S = Sample.

The friction factor under this load- and motion profile was f = 0.13 ± 0.028 (mean ± SD). Fig 7 shows the friction factor over the cycle duration.

#### 3.2.3. Subluxation.

The subluxation force for anterior subluxation with parallel endplates was 88.82 ± 4.076N (mean ± SD); and for posterior subluxation with extended endplates 77.46 ± 2.43N (mean ± SD). For lateral subluxation with parallel endplates, the subluxation force was 103.57 ± 7.59N (mean ± SD).

## 4. Discussion

The aim of the present study was to investigate whether a computationally optimized ceramic bearing for a TDR would perform adequately in terms of function and safety, also under test conditions for which it was not optimized.

The hypothesis that the investigated bearing geometry is sufficiently resistant to subluxation was confirmed. The second hypothesis, that friction parameters based on literature on ceramic-on-ceramic hip replacements could be used for the investigated TDR bearing design, could neither be confirmed nor refuted.

We conducted *in vitro* tests in a six degree of freedom universal joint simulator. We systematically determined friction parameters under multiple load- and motion profiles. We measured lower friction values than those reported for TDR bearings of other materials, but on average higher than those reported for BIOLOX®*delta-*on*-*BIOLOX®*delta* hip replacements. During all tests, no squeaking was audible. Also, no dislocations occurred, except for subluxation testing, during which dislocation was enforced. Neither the material nor the design showed any unexpected or negative behaviour.

### 4.1. Sample geometry and surface roughness

Literature has linked larger clearances to larger friction factors in hip replacements [27]. Due to large clearance, lubrication may be reduced, possibly approaching or being in boundary lubrication regime. Ceramic-on-ceramic total hip replacements have shown increased friction factors in dry conditions, compared to those tested in serum [28]. Ceramic-on-ceramic total hip replacements that had a friction factor of 0.051 ± 0.022 (mean ± SD) in serum, had a friction factor of 0.580 ± 0.079 in dry conditions, which is roughly tenfold of that in serum (48 mm diameter); for a 32 mm diameter, it was 0.096 ± 0.028 in serum and 0.491 ± 0.14 in dry conditions [28]. The radial clearance in the present study (0.24 ± 0.05 mm) was larger than that reported for the lumbar metal-on-metal TDR Maverick™ (Medtronic, USA) which has a radial clearance of 0.015 mm [6]. In ceramic-on-ceramic hip replacements, diametrical clearances are usually between 0.02–0.1 mm [29], while for metal-on-polymer and ceramic-on-polymer it is 0.16–0.4 mm [29]

In the present study, the surface roughness of the superior implant component was S_a_ = 24.5 ± 2.89 nm and S_a_ = 34.7 ± 13.1 nm for the inferior part. For the A-MAV™ (Medtronic, USA) metal-on-metal lumbar TDR, S_a_ = 9.6 ± 2.2 nm roughness was reported [30]. For BIOLOX®*delta* head it was S_a_ = 3 nm [31]. Our roughness values are considerably higher what was reported for other implants, likely because the samples of the present study were polished by hand.

The relevance of the surface roughness is that lower roughness has been connected to lower friction [32], thereby in tribological studies, the surface roughness gives an impression of how friction values relate to manufacturing and if the potential of a bearing-couple may not have been reached due to that. It might be connected to the ability to form a separating lubricant layer and with that the lubrication regime and coefficient of friction.

### 4.2. Universal joint simulator testing

In the present study, the dynamic coefficients of friction during **flexion/extension and lateral bending** were µ = 0.16 ± 0.011 (mean ± SD) and µ = 0.16 ± 0.070 (mean ± SD). Friction factors of f = 0.14 ± 0.0092 (mean ± SD) for flexion/extension and f = 0.13 ± 0.063 (mean ± SD) for lateral bending were found.

In BIOLOX®*delta-*on*-*BIOLOX®*delta* (Zimmer, USA) hip replacements, coefficients of friction of 0.14 (28 mm diameter) and 0.12 (36 mm diameter) were found after ten test repetitions [33] in bovine serum (25%). Another study, however, reported lower coefficients of friction of µ = 0.096 for smaller diameter (32 mm) and µ = 0.051 for larger diameter (48 mm) ceramic-on-ceramic hip replacement bearings ((Delta Motion®, DePuy Orthopaedics Inc., USA) in a lubricated condition [28]. Literature reports higher values for a ball-and-socket TDR made from other materials. For a cervical TDR with ball-and-socket PEEK-on-PEEK articulation (NuNec^®^, Pioneer Surgical Technology Inc., Netherlands), the friction factors for flexion were reported between 3.61–3.94 and for lateral bending between 2.67–2.83 depending on the Sommerfeld number (digitized from a graph from [18]). A study using generic ball-and-socket metal-on-metal TDR samples found friction factors in flexion between 0.29–0.89 (digitized from graphs), and furthermore showed that bearing size and applied load can affect friction factors [6].

In the present study, there is a difference between the shape of the friction loop of the lateral bending experiment compared to that of the flexion/extension experiment. This is likely caused by the difference of the shape of the trough in the corresponding directions.

The friction factor during **axial rotation** under a constant load was found to be f = 0.033 ± 0.0028 (mean ± SD). Literature on hip replacements reports a mean friction factor of 0.04 ± 0.007 for ceramic-on-ceramic (alumina-on-alumina) hip replacements in 25% serum [34] and 0.056 ± 0.01 for ceramic-on-ceramic in 100% serum [29]. Literature on the cervical PEEK-on-PEEK ball-and-socket TDR NuNec^®^ reports friction factors in axial rotation between 0.81 and 0.84 depending on the Sommerfeld number [18] (data digitized). With a friction factor below that which is reported for another TDR in literature, the presented design and material-choice are promising.

The friction factor during **coupled rotation** testing was found to be 0.13 ± 0.028 which is not in the range reported for hip replacements (44 mm head diameter) of other material pairings that had friction factors between 0.045 and 0.097 [25]. However, in that study, only part of the cycle was taken into account for the friction factor calculation. Direct comparison is strongly limited as neither the test profiles nor the sample geometry were identical to the present study. A substantial gap remains in the literature on friction factors in coupled rotation and their evaluation on TDRs

We found an anisotropy in friction factors, which is not reflected in standard finite element solvers that only use values of static and dynamic coefficients for surface couples.

The **subluxation** forces measured in this study are more than 3.5-fold the typically required values (≥ 20 N [16,35,36] or ≥ 39 N [37]). Therefore, this design can be considered resistant to subluxation. Since the TDR design is not symmetric in anterior-posterior direction, it is not surprising to find a difference between the subluxation forces in these directions. The values of the present study are within the range reported for TDRs in FDA summary of safety and effectiveness data (SSED): 22.2N - 406.9N [[16,35–38]], however, the test conditions are not necessarily identical.

In a study using BIOLOX®*delta-*on*-*BIOLOX®*delta* ball-and-socket samples representing bearings for a cervical TDR, subluxation was investigated under different conditions. Subluxations forces in the range of 64.9N - 80.9N were found [39], which is comparable to the subluxation forces of the present study of 77.46N, 88.82 and 103.57N. However, the test protocols are not identical, which limits comparison.

The accuracy of the universal joint simulator is a limitation of this study. For the load cell, it was + /- 50N for the axial force, + /- 7.5N for anterior-posterior and mediolateral forces, 0.1Nm for axial rotation and +/- 0.15Nm for flexion/extension and lateral bending. This is relevant as for example the dynamic coefficient of friction was determined as µ $=\frac{F_{F}}{F_{Ax}}$, under a 100N static axial load, the fact that the axial load is + /- 50N means that the coefficients of friction could be 0.67–2 times the value we report. While we used a correction for sensor drift and misalignment in the friction tests, in the subluxation tests, however, we did not apply a correction, as in the few tested cycles sensor drift is likely not a problem, however, misalignment may be present. The rather large radial clearance of the samples might also have affected the results. Testing on a larger number of samples would have been beneficial. It is also possible, that steady state was not reached and that the friction values would have decreased if we had tested for more cycles, overcoming sample run-in.

## 5. Conclusion

This study indicates that the computationally optimized ceramic TDR bearing is safe and can perform adequately, even under loading conditions for which it was not optimized. We report methods and results to investigate tribology and subluxation of cervical TDRs. While friction values of the samples were lower than those reported for a TDR with other bearing materials, they were comparable to or higher than for ceramic hip replacements. Based on this, it could neither be rejected nor confirmed that friction factors from hip replacements could be used for ceramic TDR bearings. Subluxation testing showed forces surpassing the acceptance criteria required in FDA SSED, which indicates that the design is sufficiently safe to subluxation.

These findings indicate that the investigated bearing design and material are suitable for TDRs. This study’s findings suggest that the investigated, design-optimized bearing may be conditionally accepted.

## Supporting information

S1 File Values behind means, SDs. (XLSX)

S2 File Values used to build graphs. (XLSX)
